# Comparative (Computational) Analysis of the DNA Methylation Status of Trinucleotide Repeat Expansion Diseases

**DOI:** 10.1155/2013/689798

**Published:** 2013-12-23

**Authors:** Mohammadmersad Ghorbani, Simon J. E. Taylor, Mark A. Pook, Annette Payne

**Affiliations:** ^1^Department of Information Systems and Computing, Brunel University, Uxbridge Middlesex UB8 3PH, UK; ^2^Division of Biosciences, School of Health Sciences & Social Care, Brunel University, Uxbridge Middlesex UB8 3PH, UK

## Abstract

Previous studies have examined DNA methylation in different trinucleotide repeat diseases. We have combined this data and used a pattern searching algorithm to identify motifs in the DNA surrounding aberrantly methylated CpGs found in the DNA of patients with one of the three trinucleotide repeat (TNR) expansion diseases: fragile X syndrome (FRAXA), myotonic dystrophy type I (DM1), or Friedreich's ataxia (FRDA). We examined sequences surrounding both the variably methylated (VM) CpGs, which are hypermethylated in patients compared with unaffected controls, and the nonvariably methylated CpGs which remain either always methylated (AM) or never methylated (NM) in both patients and controls. Using the J48 algorithm of WEKA analysis, we identified that two patterns are all that is necessary to classify our three regions CCGG∗ which is found in VM and not in AM regions and AATT∗ which distinguished between NM and VM + AM using proportional frequency. Furthermore, comparing our software with MEME software, we have demonstrated that our software identifies more patterns than MEME in these short DNA sequences. Thus, we present evidence that the DNA sequence surrounding CpG can influence its susceptibility to be *de novo* methylated in a disease state associated with a trinucleotide repeat.

## 1. Introduction

**Figure 1:**
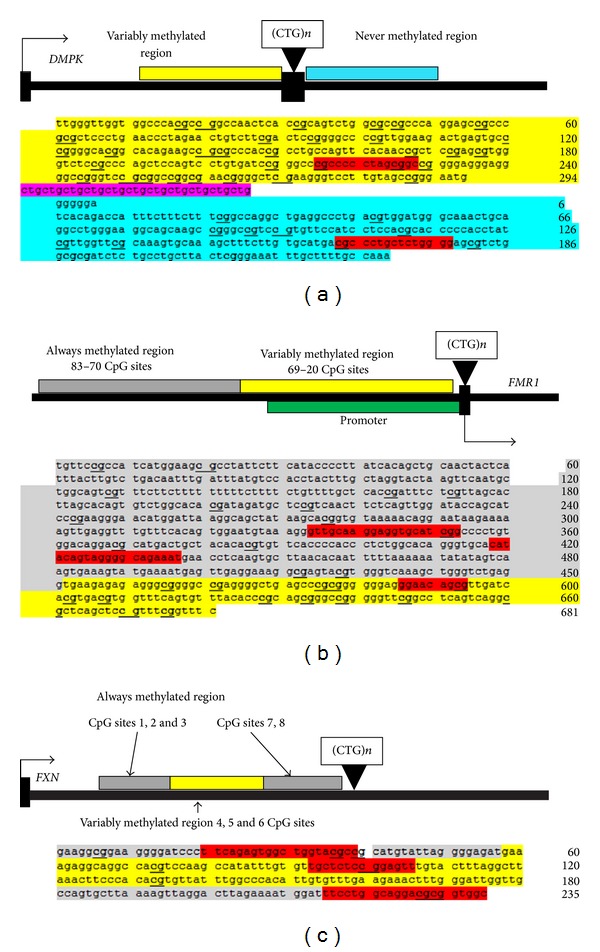
This figure shows the 3 gene regions under investigation: (a) *DMPK* 3′ UTR region, (b) *FMR1* 5′ UTR region, and (c) *FXN* intron 1 region. A scheme of the DNA sequence, transcriptional start site, and the regions analysed are shown. The grey shading shows the always methylated (AM) regions, blue shows the never methylated (NM), and the yellow area shows variably methylated (VM) regions. CpG sites are underlined and bold numbers at start and end of each line show base pair number in the sequence. A triangle shows the location of repeat expansion and the box above triangle shows the TNR repeats. The green highlighted region in the *FMR1* gene indicates the promoter region. The CTCF binding sites are shown in red.

DNA methylation involving the addition of a methyl group to a CpG sequence is one of the mechanisms of gene regulation commonly associated with transcriptional repression and is necessary for mammalian development, X inactivation, and genomic imprinting [1]. Gene silencing is a major biological consequence of DNA methylation. The phenomenon is widely reported in genes of both healthy cells, where it assists in regulating gene expression during development, for example, and diseased cells, where it is associated with aberrant gene expression most notably in cancerous cells. One group of diseases in which DNA methylation is reported to have an important role is TNR expansion diseases. Here, we investigate the pattern of sequences in variably methylated (VM) and nonvariably methylated (AM always methylated and NM never methylated) CpG sites of three TNR expansion diseases: FRDA, FRAXA, and DM1 [2]. FRDA is an inherited autosomal recessive neurodegenerative disorder characterised by a homozygous GAA repeat expansion within intron 1 sequence of the *FXN* gene [3]. The consequence of the expanded GAA repeats is to reduce the expression of the mitochondrial protein frataxin. Typically unaffected individuals have 5–32 GAA repeats and affected individuals have 66–1700 repeats. FRAXA is a mental retardation disorder associated with one of the seven folate-sensitive fragile sites that have been identified within human chromosomes. All of these sites have a large noncoding CGG repeat expansions. FRAXA is the most prominent of the fragile site disorders [4]. It is caused by repeat expansion with 5′ UTR of the *FMR1* (fragile X mental retardation 1) gene [5]. Unaffected individuals have 6–55 CGG repeats and affected individuals have 55–200 repeats [6]. DM1 is an autosomal dominant disorder which is characterised by clinical features such as muscle weakness, myotonia, and heart defects [7]. DM1 is characterised by expansion of CTG repeats within the 3′-UTR of the *DMPK* gene [8]. Unaffected individuals have 5–37 CTG repeats and affected individuals have 90 to several thousand CTG repeats [9]. These are currently the only trinucleotide repeat diseases that have been studied with respect to their DNA methylation patterns near the trinucleotide repeats [2]. All the DNA methylation data and details of the patients and controls used are prepublished in references 38, 39, and 40. All the CpG sites studied are very close to the repeat and therefore have the potential to be methylated in patients due to the repeat expansion. However, they are not all methylated, as shown in Figure 1. The proximity of the CpG to the repeat does not seem to be the only factor influencing aberrant methylation in patients as demonstrated by the *FXN* gene, where CpGs that are nearer to the repeat do not show variability in methylation between patients and controls.

A common theme for all of the large noncoding TNR expansion diseases is DNA hypermethylation of CpG dinucleotides near the repeats (3). The mechanism by which aberrant methylation occurs is poorly understood, but several theories have been put forward. Short interfering RNAs (siRNAs) produced by bidirectional transcription across TNRs may recruit histone methyltransferases, HP1, and DNA methyltransferases giving rise to DNA methylation [10]. siRNAs have been shown to be produced from gene promoter CpG islands [11] and at several TNR loci [12–16] thus making this mechanism possible. Further, it is possible that siRNAs generated from a different locus may induce DNA methylation at a TNR locus. Loss of a methylation-sensitive chromatin insulator and subsequent spreading of DNA methylation might be another mechanism for DNA methylation at TNR loci. The insulator protein CTCF is one possible candidate for this mechanism because it has been identified in the flanking regions of FRAXA CGG repeats [14], DM1 CTG repeats [17], and also in the upstream regions of FRDA GAA repeats [15].

DNA methylation in TNR expansion disorders is thought to result in the silencing of gene transcription by two general mechanisms (i) preventing binding of basal transcription protein or other regulatory DNA binding proteins (e.g., CTCF) and (ii) influencing nucleosome positioning or stability and reinforcing heterochromatin formation through the actions of methyl CpG binding proteins (MBPs), histone modifications, and chromatin remodelling [18]. TNR expansion disorders characteristically show trinucleotide instability which plays a significant role in the progression and aetiology of the disease. DNA methylation in these diseases appears to influence the dynamics of that stability [9]. Thus, DNA methylation is not only a mechanism by which the disease is caused but a mechanism by which it develops.

Particular motifs have been identified which predict the methylation status of DNA sequences in normal cells. Notably methylation is more prevalent in regions of low CpG density, with regions of intermediate density being most variably methylated [19]. Computational methods have also been used to show that the frequencies of CpG, TpG, and CpA are different between unmethylated and methylated CpG islands [20]. Further, Yamada and Satou [21] used machine learning by support vector machine and random forest using previously reported methylation data to analyse DNA sequence features to predict methylation status. They revealed that frequencies of sequences containing CG, CT, or CA are different between unmethylated and methylated CpG islands. Ali and Seker [22] used an adapted K-nearest neighbour classifier to predict the methylation state on chromosomes 6, 20, and 22 in various tissues. They identified four feature subsets which had shown that the methylated CpG islands can be distinguished from the unmethylated CpG islands. Lastly, Previti et al. [23] used a mining process in the absence of supervised data to cluster and then predict methylation status of CpG islands in different tissues and showed significant differences in the sequences of CpG islands (CGIs) that predispose them to such methylation. In their review of computational epigenetics, Bock and Lengauer [20] in their review “Computational Epigenetics” highlighted the fact that, although it is clear that much work has been done to document the epigenetic state of the genome (much of it reported in the ENCODE project (http://www.nature.com/encode/#/threads)), work in the area of *de novo* DNA methylation prediction is to date limited. Aberrant methylation has been shown to be associated with mutations. Methylation in the MGMT promoter has been demonstrated to be closely associated with G : C to A : T mutations [19]. A few studies have attempted to search for motifs associated with aberrant methylation most notably Feltus et al. [24] who used Restriction Landmark Genome Scanning software to identify methylation resistant and methylation prone motifs based on DNA sequence. Their results suggest that the sequence surrounding a CpG can be used to predict aberrant methylation. In another study by McCabe et al. [25], patterns were identified using machine learning techniques and used for pattern matching. DNA signatures and a cooccurrence with polycomb binding were found to predict aberrant CpG methylation in cancer cells.

Previous studies have examined DNA methylation in different trinucleotide repeat diseases [26–28]. In this study, we have used a pattern searching algorithm on the combined data from these studies to identify motifs in the DNA surrounding aberrantly methylated CpGs found in the DNA of patients with one of the three TNR expansion diseases: FRAXA, DM1, or FRDA. We examined sequences surrounding both the variably methylated (VM) CpGs, which are hypermethylated in patients compared with unaffected controls, and the nonvariably methylated CpGs which remain either fully methylated (AM) or unmethylated (NM) in both patients and unaffected controls. We expand on the approach of Lu et al. [29] using a search window of 5 bp allowing up to 3 mismatches. Any sequence with 4 mismatches is discounted because they represent only a one bp motif. Patterns identified therefore include mismatches. For example, if the sequence contains GATCT, it is counted in GA**T, GA*CT, where ∗ represents any bp in the motif.

## 2. Materials and Methods

### 2.1. Pattern Generation

The three DNA sequences of *FXN* [26], *FMR1* [27], and *DMPK* [28] genes involved in the 3 TNR expansion diseases were examined for methylation status in patients and controls in previous studies. The methylation results obtained in these previous studies were used as the data for our work. CpG sites for which their methylation statuses were available for both disease and normal cells were tagged. In order to identify patterns in the sequences flanking these CpGs, all possible sequences of a window size of 5 bp were generated in similar way to those used in the study by Lu et al. [29]. DNA of 5 bp length has been shown in the literature to have significant roles in biological functions. For example, some of them are modifying sites and binding sites of enzymes and some are binding motifs of some transcription factors. It has also been shown that 5 bp DNA lengths are important for DNA methylation where they are probably associated with the binding of DNA methyltransferase [29]. 5 bp long sequences are important for the binding of many enzymes including the methyltransferase; both methylases (*Lla*DII and *Bsp*6I R/M) have two recognition sites (5′-GCGGC-3′ and 5′-GCCGC-3′) [3-0] and 5′-CCCGC-3′ is the recognition site of the DNA methyltransferase (methylase) FauIA (of the restriction-modification system FauI from *Flavobacterium aquatile*) [30].

Sequences from *FXN*,* FMR1*, and *DMPK* genes were divided into three classes of region, always methylated (AM), variably methylated (VM), and never methylated (NM), where the regions that are variably methylated are aberrantly methylated in patients and the always and never methylated regions are methylated and nonmethylated, respectively, in both patients and controls. Significantly, there is no overlap between regions. This is shown in Figure 1. Both positive and negative strands were analysed. For each of these regions, the 5 bp window “slides along” from start to end of sequence and the pattern in that window is noted plus some additional information, the CpG site that patterns occurred near, location of pattern (using numbering as shown in Figure 1) and the exact sequence that occurred in the window (see Table 1 in Supplementary Material available online on http://dx.doi.org/10.1155/2013/689798).

The patterns identified were ranked in order of frequency in a region class and the proportional frequency in each region as calculated by dividing the frequency in that region by the length of the region in bp. The proportional frequencies of each pattern in each region class were calculated by adding all the regions in that class together giving the sum of that proportional frequency in that region class. Thus we are able to determine which patterns are most prevalent in each methylation region class. We were able to identify patterns that are not present in any one class and more prevalent in the other two classes using the sum of the other two classes' proportional frequencies (Table 1) and also patterns that are unique in one class and did not occur in the other two classes (Table 2). This allowed us to determine which pattern(s) best discriminated between the region classes (Supplementary Table 1).

Further, to validate and compare our results, we used MEME software (http://meme.nbcr.net/meme/) to identify patterns in these same regions. A 5 bp window size was used and “any number of repetitions” was selected; all other settings were default.

The WEKA J48 classification technique was used to find the patterns that best classify the sequences in the three classes. The patterns of each region were used as attributes in the analysis rather than the sums of all the regions in the same class. The patterns were treated as attributes in WEKA and sequences as instances. We used the WEKA J48 algorithm (an implementation of the C4.5 algorithm) to generate a decision tree. We used Witten et al.'s approach to implement the decision tree to classify the pattern as follows. “First, select an attribute to place at the root node, and make one branch for each possible value. This splits up the example set into subsets, one for every value of the attribute. Now, the process can be repeated recursively for each branch, using only those instances that actually reach the branch. If at any time all instances at a node have the same classification, stop developing that part of the tree” [31]. Attributes are selected based on information gain, so in our tree CCGG* has the highest information gain.

To determine if any of the patterns have an identity to known DNA motifs for such DNA binding proteins as transcription factors we analysed the patterns identified by WEKA as distinguishing each region class using TOMTOM software (http://meme.nbcr.net/meme/) using the Jasper and UniPROBE databases for the TOMTOM search.

## 3. Results

### 3.1. Frequency

Of all the possible combinations of 5 bp patterns where 2 or more of the 5 bps are identical within a pattern for example, CG*** is one pattern where patterns of CGTTG and CGTTA are the actual sequences found. 1584 different patterns were found in all the regions analysed. Most were found in all 3 genes in all the regions. 1454 patterns were found in the VM regions, 1563 in the AM regions and 1264 in the NM region. Two patterns are unique in the *FMR1* gene in both regions. There are no unique patterns for *FXN*. One pattern is unique for the *DMPK* gene in both regions. Analysis of the patterns revealed that some patterns did not occur in some regions allowing the region classes to be separated; these results are shown in Table 1.

### 3.2. Proportional Frequency

On calculating the sum of the proportional frequencies of patterns, we found three patterns are unique for VM regions and 84 are unique to AM. There are no unique patterns for the NM region. The patterns which showed the greatest proportional difference between the regions are given below and show that there are patterns which are unique to VM and AM regions. Results are shown in Table 2.

The summed proportional frequencies of each pattern for each region class showed a distinct difference in the frequencies of particular patterns in different class regions. Our results clearly show that some patterns are more prevalent in some region classes than others and therefore the methylation status of the regions around the repeats is influenced by the underlying DNA sequence as well as the length of the trinucleotide repeat.

### 3.3. WEKA Analysis

**Figure 2:**
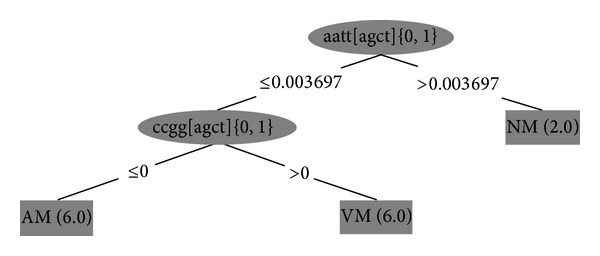
Decision tree created by Weka package. AM is always methylated, NM is never methylated (NM) and (VM) is variably methylated.

The finding from the frequencies showing that some patterns could be used to distinguish the 3 class regions from each other was confirmed by J48 classification decision tree analysis using WEKA software. The results are given in Figure 2. The WEKA programme identified that two patterns are all that is necessary to classify our 3 regions, as shown in the decision tree.

Using the proportional frequencies of all regions (not the summed frequencies), we observed that AATT* distinguished between NM and VM + AM using the J48 algorithm where the proportional frequency of AATT* is more than 0.003697 in NM. This result mirrors the result of the frequency analysis reported above that there are no unique patterns for the NM region; hence, the distinction is based on frequency rather than the presence or absence of a pattern. AM can be distinguished from VM by the sequence CCGG* which is found in VM and not in AM regions.

### 3.4. Comparison with MEME Software

In order to compare our software's predictions to those generated by MEME, we compared the 10 best distinguishing motifs identified by MEME, using the any number of repetitions (ANR) option and a window size of 5 bp, similar to our algorithm, with the patterns identified by our software. The results are given in Table 3. The results are comparable, but notably our software identified more patterns than the MEME software. No patterns found using the MEME software were missed by our software.

### 3.5. CTCF Binding

Since it has been reported that FRDA patients have depleted levels of CTCF and there is a suggestion that this protein could act to protect DNA from targeted methylation in healthy individuals [15], the regions were analysed for CTCF binding sites to determine if the differential methylation could be linked not only to DNA sequence but also to CTCF binding. The diagrams in Figure 1 show the putative CTCF binding sites in the analysed regions. Since the bindings sites seem to be equally prevalent in all the region classes, it would seem that simple depletion of CTCF levels may not be the explanation for the variability in the methylation in patients compared to controls unless there are other factors that influence the binding of CTCF to its site over and above just the binding site sequence. Proof that CTCF may not be the complete explanation of differential methylation in patients requires wet laboratory experiments that are beyond the scope of this paper.

### 3.6. Comparison of Patterns with DNA Binding Protein Sites

Hogart et al. [32] identified overrepresented transcription factor consensus binding motifs in methylated sequences. This would suggest that the methylation-sensitive binding of DNA binding proteins plays an important part in regulating genes. Thus, the variation in methylation seen in VM regions could be an important mechanism in these disease states due to the DNA binding proteins that bind to these regions. Further, since the binding of DNA binding proteins such as transcription factors may influence aberrant methylation patterns, we wished to compare our patterns with binding sites in the Jasper and Uniprobe databases. TOMTOM analysis revealed that the CCGG pattern which is found in VM regions but not AM regions is part of the consensus binding site for 35 DNA binding proteins; however, not all are found in mammals. A human protein ELK4, which is predicted to bind, may be influenced by the degree of methylation in the promoter of some genes as demonstrated in the caldesmon gene (*CALD1*) by Cooper et al. [33]. Another one, GABPA, whose binding sites are overrepresented in methylated regions of primary mouse hematopoietic stem cells [32], shows evidence of GABPA binding being methylation-sensitive as demonstrated by Lucas et al. [34] who showed that the regulation of TMS1/ASC gene is controlled in such a way.

The AATT pattern which is found more frequently in NM matched with 151 DNA binding protein consensus sequences, although very few are found in mammals. However, there was a preponderance of homeobox domain proteins in the matches. One protein predicted to bind, PAX6, is inhibited from binding by methylation of its binding site [35]. Another one, PAX7, results in H3 K4 trimethylation of surrounding chromatin stimulating transcriptional activation of target genes to regulate entry into the myogenic developmental programme in skeletal muscle [36]. There is therefore a suggestion that these patterns may bind proteins that could influence gene expression. Wet laboratory experiments are however required to prove that this is indeed the case.

## 4. Discussion

Our results show that there are sequence patterns which can be used to distinguish between AM, VM, and NM regions of these TNR genes. A single pattern can be used to distinguish the NM region from the other two. Furthermore, the fact that the VM regions show a few striking and unique patterns is particularly notable when the frequencies are summed and WEKA analysis of nonsummed frequencies shows that one pattern can be used to distinguish this region class from AM. This finding could point towards one mechanism which contributes to the methylation status of these regions of DNA in patients compared with controls.

The three genes however show differences based on our classification of the VM, AM, and NM regions. There could be several explanations for this: for *DMPK*, the VM region is upstream of the TNR and has the only NM region in any of the genes which is downstream of TNR. In *FMR1* and *FXN*, both VM and AM regions are upstream of the repeat region. *DMPK* and *FMR1* are similar in the way that their AM region is continuous unlike *FXN* which has two disconnected AM regions. Further, the nature of the TNRs in each of the genes is different; *FXN* has a TNR of GAAn, *FMR1* has CGGn, and *DMPK* has CTGn. Thus, *FXN* is unique in having only purines in one strand of its repeat (and only pyrimidines in the other strand), while the other two repeats are mixtures of purines and pyrimidines in each strand.

Comparing our software to identify patterns with MEME, we have demonstrated that our software identifies more patterns than MEME in these short DNA sequences. MEME software has been optimised to find patterns in much longer sequences thus may not be as good as our software for detecting patterns in short sequences or using small window sizes. Further, when the results from MEME alone on our sequences were analysed using WEKA (see supplementary data), the software gave a less discriminating tree than the results from our software, thus showing our software is better at discriminating patterns than MEME.

There are many possible points of discussion that can be drawn from our data. The report that FRDA patients have depleted levels of CTCF suggested the possibility that this protein could act to protect DNA from targeted methylation in healthy individuals [15]. However, the distribution of potential CTCF binding sites in the three genes examined here would suggest that this is not the sole cause of the variation in methylation seen in the different regions.

It is notable that the restriction sites of the two classical enzyme pairs *Hpa*II—*Msp*I (CCGG) and *Sma*I—*Xma*I (CCCGGG) used to analyse DNA sequences for methylation have CCGG at their core. Although not all CpG methylation occurs within these sequences, much does. This illustrates the significance of discovering the CCGG pattern as a mark for VM regions.

DNA methyltransferases 3a and 3b (Dnmt3a or Dnmt3b) are the enzymes responsible for *de novo* DNA methylation in humans and the mouse. However, the mechanisms by which specific DNA sequences are targeted to be methylated are not known, nor are the signals that trigger this phenomenon. The work of Hervouet et al. [37] has shown that Dnmt3a and Dnmt3b have consensus sequences to methylate DNA (T/A/C)(A/T)(T/G/A)CG(T/G/C)G(G/C/A) and (A/C)(C/G/A)(A/G)CGT(C/G)(A/G). Thus, the propensity of a methylase to *de novo* methylate certain CpG may not happen due to the binding specificity of the enzyme itself since these sequences demonstrate the low specificity of these enzymes. Hervouet et al. go on to suggest that the mechanism is controlled by the interaction of Dnmt3a or Dnmt3b with specific transcription factors suggesting that the specificity comes from the binding or not of these transcription factors to specific sequences in the promoter regions of genes. It is also possible that there is an interaction of antisense RNA with specific DNA sequences or with the methylases themselves to molecules that may aid in the directing of *de novo* methylation. Epi-miRNAs have been demonstrated to regulate or possibly direct the epigenetic machinery as reported in a review by Iorio et al. [38]. Either of these mechanisms could lead to the more directed *de novo* methylation seen *in vivo* and therefore could explain the differences between the logos characterised for the 3 different genes investigated in this work.

Such aberrant methylation is well known to cause downregulation of genes resulting in disease states by very different mechanisms. In cancer, the aberrant methylation is not under the influence of TNRs present near the genes; thus, the mechanism giving the observed variation in methylation in these genes is probably subtly different. Furthermore, the resulting methylation may result in different effects. In *FMR1*, DNA methylation prevents the binding of the transcription factor *α*-Pal/NRF-1, whereas methylation of the *FXN* intron 1 region may be involved in the formation of a transient purine ·purine·pyrimidine DNA triplex preventing transcriptional elongation [39]. Recently microRNAs have been hypothesised to have a role in the downregulation of genes. It has been shown that microRNA expression can be modulated by promoter methylation or histone acetylation, a phenomenon that is found in numerous diseases including FRDA. Also antisense RNAs may be more highly expressed. Interestingly, the work by De Biase et al. [15] shows the presence of increased amounts of a novel transcript *FAST-1* (*FXN* antisense transcript-1) in FRDA patients which may prove to be significant.

Thus, we present evidence that the DNA sequence surrounding a CpG can influence its susceptibility to be *de novo* methylated in a disease state associated with a trinucleotide repeat and that our work could form the basis of directed wet laboratory experiments to prove the phenomenon. This supports the findings of other investigators who have made similar findings in cancer cells [25]. Our results represent those from only three of the numerous trinucleotide repeat associated diseases since data for the other diseases is unavailable at this time. We acknowledge therefore that further work to elucidate the involvement of DNA methylation and then the DNA sequence around any methylated CpG islands in patients is required to build a complete picture of this phenomenon in this classification of diseases.

## Supplementary Material

Supplementary material Table 1 shows the all the patterns identified and their location for each CpG site and region. The exact sequence is given as well as the patterns.

## Figures and Tables

**Table 1:** Table of discriminatory patterns.

Top 10 patterns that separate AM class from NM and AM (less frequent in AM than NM + VM)	Top 10 patterns that separate VM class from AM and NM (less frequent in VM than AM + NM)	Top 10 patterns that separate NM class from AM and VM (less frequent in NM than AM + VM)
ccgg[agct]{0,1}	[agct]{0,1}tttt	ta[agct]{0,2}a
g[agct]{0,1}gcg	t[agct]{0,1}cat	[agct]{0,1}gcgg
g[agct]{0,1}ctc	catg[agct]{0,1}	[agct]{0,1}ccgc
cgg[agct]{0,1}t	ga[agct]{0,1}at	c[agct]{0,1}ccg
ag[agct]{0,1}ct	at[agct]{0,1}ca	ag[agct]{0,1}gg
cg[agct]{0,1}tc	taa[agct]{0,1}t	c[agct]{0,1}gcg
cga[agct]{0,1}c	tct[agct]{0,1}a	ag[agct]{0,1}ac
t[agct]{0,1}cga	[agct]{0,1}tgca	tt[agct]{0,1}aa
gt[agct]{0,1}ac	ta[agct]{0,1}ta	ctt[agct]{0,1}a
tcga[agct]{0,1}	ac[agct]{0,1}ta	ttt[agct]{0,1}a

**Table:** (a) Patterns unique to VM

Pattern	Sum (VM)	Sum (AM)	Sum (NM)
ga[agct]{0,1}tc	0.006803	0	0
g[agct]{0,1}gac	0.010544	0	0
gt[agct]{0,1}ac	0.014286	0	0

**Table:** (b) Top 10 proportionally most frequently occurring patterns unique to AM

Pattern	Sum (VM)	Sum (AM)	Sum (NM)
taa[agct]{0,1}t	0	0.043757	0
tct[agct]{0,1}a	0	0.043119	0
ta[agct]{0,1}ta	0	0.038785	0
ga[agct]{0,1}aa	0	0.034818	0
ta[agct]{0,1}at	0	0.030483	0
t[agct]{0,1}tat	0	0.028634	0
at[agct]{0,1}ag	0	0.026786	0
[agct]{0,1}atac	0	0.024938	0
a[agct]{0,1}tag	0	0.024938	0
tat[agct]{0,1}a	0	0.024938	0

**Table:** (c) The 8 proportionally most frequently occurring patterns which are more prevalent in NM than in VM and AM

Pattern	Sum (VM)	Sum (AM)	Sum (NM)
[agct]{0,1}atct	0	0.001848	0.004348
agat[agct]{0,1}	0	0.001848	0.004348
a[agct]{0,1}gat	0	0.003697	0.008696
atc[agct]{0,1}t	0	0.003697	0.008696
[agct]{0,1}atcg	0	0.005545	0.008696
cgat[agct]{0,1}	0	0.005545	0.008696
atcg[agct]{0,1}	0	0.005545	0.008696
[agct]{0,1}cgat	0	0.005545	0.008696

**Table:** (a) 10 best 5 bp motifs in variably methylated regions

Pattern	MEME detail positive or negative strand	Our software variably methylated
TGTTT	FXN+, FMR1+	FMR1+, FXN+
AAACT	FXN++−	FXN++−
TATTT	FXN++	FXN++
TCCAA	FXN+DMPK−	FXN+DMPK+
TCGAA	DMPK+DMPK−	DMPK+DMPK−
**CTGAG**	**FMR1 − −**	**DMPK+FMR1+FMR1− −**
CTGAA	FMR1−DMPK+	DMPK+FMR1−
**GAGAG**	**FXN−FMR1**+	**FMR1++FXN**− −
TA[CG]AA	DMPK−DMPK+	DMPK−DMPK+FXN−
ACCCA	DMPK− −	DMPK − −

**Table:** (b) 10 best 5 bp motifs in always methylated regions

Pattern	MEME detail positive or negative strand	Our software always methylated
AGGGG	FXN_AM_1++FMR1+−−	FMR1+−−FXN_AM_1++
**CCAGC**	**FXN_AM_1−FMR1−**	**FMR1**+−−**FXN_AM_1−**
**CTGGC**	**FXN_AM_2+FMR1+**	**FMR1**+++**FXN+**
**CCACC**	**FXN_AM_2−FMR1+**	**FMR1**++**FXN_AM_2−**
**CCTCA**	**FMR1**− −	**FMR1+**− − −
CCGCC	FXN_AM_1−FMR1+	FXN_AM_1−FMR1+
**AGCAC**	**FXN_AM_2−FMR1−**	**FXN_AM_2−FMR1+++−**
**AGTTG**	**FMR1++**	**FMR1+++FMR1**− −
**TAGCA**	**FMR1−FMR+**	**FMR1**++ − −
AGAAA	FXN_AM_2+FMR1++−−− −	FXN_AM_2+FMR1++−−− −

**Table:** (c) 10 best 5 bp motifs in never methylated region

Pattern	MEME detail positive or negative strand	Our software never methylated
**TTTGC**	**DMPK++−**	**DMPK**++−−−
**TTCTT**	**DMPK++**	**DMPK+++**
AGGCA	DMPK−+	DMPK+−
**TT[AT]CT**	**DMPK**++	**DMPK++++**
CCATC	DMPK+−	DMPK+−
**CAGGC**	**DMPK++**	**DMPK**++−−
CAGAC	DMPK−+	DMPK+−
TGACG	DMPK++	DMPK++
ACC[AT]A	DMPK−+	DMPK+−
CTGGG	DMPK++	DMPK++

Key:

+ Positive strand.

− Negative strand.

FXN_AM_1 and FXN_AM_2 are two separated regions with always methylated CpG sites.

**Bold**: Motif that MEME does not report all occurrences.
